# Impact of virtual reality on peri-interventional pain, anxiety and distress in a pediatric oncology outpatient clinic: a randomized controlled trial

**DOI:** 10.1186/s12887-024-04952-3

**Published:** 2024-08-03

**Authors:** Alicia Reitze, Marie Voigt, Frank Klawonn, Martin Dusch, Lorenz Grigull, Urs Mücke

**Affiliations:** 1 Hannover Medical School, Pediatric Oncology and Hematology, Hanover, Germany; 2 Helmholtz Centre for Infection Research, Braunschweig, Germany; 3 Ostfalia University, Wolfenbüttel, Germany; 4 Hannover Medical School, Anesthesiology and Intensive Care Medicine, Hanover, Germany; 5 University Hospital Bonn, Center for Rare Diseases, Bonn, Germany

**Keywords:** Virtual reality, Pediatric oncology, Outpatient clinic, Peri-interventional pain

## Abstract

**Purpose:**

Pain and anxiety-inducing interventions have a major impact on pediatric patients. Pain reduction by virtual reality (VR) during port and vein punctures is well studied. This study investigates peri-interventional reduction of pain, anxiety and distress using VR compared to the standard of care (SOC) in a pediatric oncology outpatient clinic.

**Methods:**

In a randomized, controlled cross-over design, patients aged 6–18 years experience potentially painful interventions accompanied by VR. Observational instruments include NRS, FPS-r, BAADS, mYPAS-SF, PedsQL and SSKJ3-8R. All patients undergo two observations: SOC (A) and VR (B) in a randomized order. In addition, parents and staff are interviewed. Specific conditions for VR in an outpatient clinic setting derived from interprofessional focus group discussion are being explored.

**Results:**

Between July 2021 and December 2022 57 eligible patients were included and randomized to the orders A/B (*n* = 28) and B/A (*n* = 29). Thirty-eight patients completed both observations. Characteristics in both groups did not differ significantly. More than half of the patients had no previous experience with VR, 5% decided to discontinue VR prematurely. Peri-interventional pain, anxiety and distress were significantly reduced by VR compared with SOC. 71% of patients and 76% of parents perceived punctures with VR to be more relaxed than previous ones. 95% of patients perceived fun with VR goggles. Detailed questionnaires on individual stress and anxiety were returned from 26 of 38 patients. Focus group discussion with staff yielded evidence for successful implementation of VR in an outpatient clinic.

**Conclusions:**

The present study shows that VR can be used for peri-interventional reduction of pain, anxiety, and distress in the special environment of a pediatric outpatient clinic. Specific conditions must be met for successful implementation. Further studies are needed to identify particularly susceptible patients and to illuminate alternatives for distraction that are feasible to implement with limited resources.

**Trial registration number:**

(ClinicalTrials.gov ID): NCT06235723; 01/02/2024; retrospectively registered. This study adheres to the standard checklist of CONSORT guidelines.

## Key messages

***What is known***

• Interventions in pediatrics cause anxiety, distress and pain.

• Pain-reducing methods require specific conditions in the context of an outpatient clinic.

• Virtual reality has the potential to reduce anxiety and pain in addition to other methods.

***What is new***

• VR enjoys great popularity among pediatric oncology patients, parents, and staff.

• Self- and peer-assessment tools for pain, anxiety, and stress can be used for intra- and inter-individual comparison.

• For the implementation of VR to succeed in an outpatient clinic, specific conditions need to be taken into account.

• Questionnaires could be used to identify particularly susceptible patients in advance.

## Introduction

Potentially painful and stressful interventions limit quality of life in pediatrics. Especially in pediatric oncology, invasive procedures such as lumbar and bone marrow punctures occur regularly as part of diagnosis and therapy. Particularly in the case of leukemia, which accounts for about one-third of all childhood cancers, regular punctures are part of the patients’ everyday life [1]. Also, for hematological diseases punctures must be performed frequently. Standard approaches such as local anesthetic cream and analgesia can help reduce pain, but they do not affect anxiety and distress directly. Peri-interventional effective methods are often lacking. Reasons are, for example, lack of personnel, insufficient training, ignorance of possibilities, lack of technical prerequisites, time and knowledge about individual needs, and deficits in interprofessional cooperation [2]. Distraction as a method of stress and pain reduction is often known and attempted through conversation, smartphones, or books [3]. While virtual reality (VR) has been investigated for pain reduction e.g. in wound care and intravenous access, there is a lack of evidence on the effect of VR in the specific setting of an oncology outpatient clinic and the necessary conditions for its application [4, 5]. In particular, studies on susceptible patients considering adverse effects and necessary implementation steps in resource-limited settings are relevant. The results and methodological quality of previous studies on VR in pediatric oncology are heterogeneous. Recent work suggests the potential of VR as a complementary intervention [6]. In this study, we compare the peri-interventional use of VR with the individual standard of care (SOC) in terms of reduction in pain, anxiety and distress. Additionally, observations from patients’, parents’ and staffs’ perspective are discussed concerning successful implementation.

## Materials and methods

### Study design and sample

The present study was a monocentric, prospective, randomized, open-label, cross-over investigation. It was conducted at Hannover Medical School from July 2021 to December 2022.

Patients aged 6–18 years, regardless of sex, who underwent a painful puncture (port puncture/placement of a peripheral venous catheter) or a stressful intervention (lumbar puncture/bone marrow puncture) using their Broviac catheter, were included. Patients without a painful puncture were included in all analyses except for the assessment of pain levels. Detailed informed consent was obtained from patients and guardians for study participation. Exclusion criteria were epilepsy, coronary artery disease, history of severe vertigo, obstacles to putting on and wearing VR glasses, and lack of informed consent for study participation. Review of inclusion and exclusion criteria was performed by the study team during the week prior to each appointment at the outpatient clinic. The study was approved by the local ethics committee.

### Study setting

The setting of the study represents a pediatric oncology outpatient clinic where patients are examined and undergo punctures or other potential painful procedures in an intervention room. The treatment is provided by nursing staff and physicians of different levels of experience. Depending on the number of patients and workload, the opportunities to provide detailed guidance to patients vary widely in terms of individual stress and anxiety reduction. There are other known technical possibilities for distraction besides VR such as the use of smartphones.

### Randomization

Sample size calculation was based on the Wilcoxon-Mann-Whitney test. The calculation was performed with a defensively rounded effect size of 0.8 [7], an α-error of 0.05, and a power of 0.95. This resulted in a sample size of *n* = 37 per group. After consent was obtained, patients were randomized into an order for use of the distraction methods. Thus, patients received distraction by VR peri-interventionally (B), then according to their standard of care (A), or in reverse order (A/B).

### Virtual reality

Virtual reality was applied with the help of goggles. These were demonstrated to the patients, then put on after entering the room. Patients were able to choose between three passive distraction videos. Active application (e.g. games) was not used due to the necessary interventions avoiding sudden movements. The goggles could be taken off at any time. The time of VR usage, reasons for any discontinuation, and further adverse reactions were documented.

### Standard of care

The individual standard of care was determined by the patients themselves. In addition to the choice of distraction method, this also included the optional use of local anesthetic cream on the potential puncture site for port or vein puncture. The respective choice was documented. Detailed observations on individually selected methods during the pre-interventional waiting period were not recorded.

### Data collection and assessment tools

Demographic data and prior experience information were collected prior to the first intervention. Specific aspects of stress management and health related quality of life were collected with help of SSKJ3-8R and PedsQL [8–10]. Different tools for assessing pain, anxiety, and distress were used before, during, and after the painful interventions. Specifically, the following tools were used: NRS (for patients of ten years and older) and FPS-r (for patients of nine years and younger) for the assessment of pain, mYPAS-SF for the measurement of anxiety and BAADS for distress assessment [11–14]. After the intervention, assessments of enjoyment, likelihood of repetition, and observations of use by parents and staff were recorded. After data collection was finished an interprofessional focus group with involved staff members was used to facilitate conditions for successful implementation of VR. The five participants (nurses and doctors) were asked to describe their experiences with VR during the study. Afterwards, they were asked to share and discuss personal tips that would be helpful for successful implementation. The discussion was moderated by an investigator and summarized based on the 10 tips formulated by consensus.

### Data analysis

Data preparation and analysis were performed with SPSS version 27 and R version 4.2.3. Characteristics of the study population were described through methods of descriptive statistics. Comparison of groups was carried out with the t-test for independent samples.

## Results

**Fig. 1:**
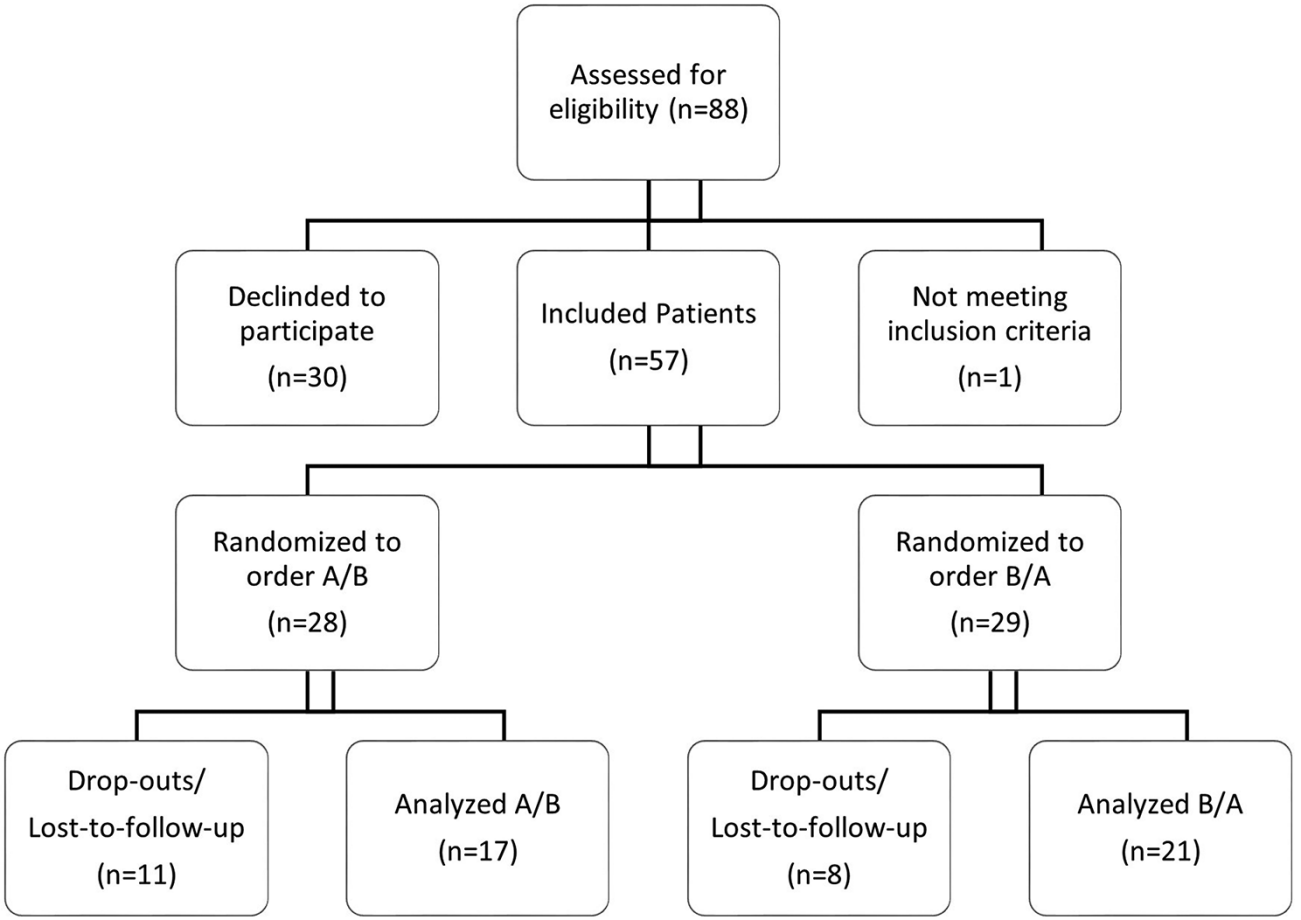
Participant flow diagram of all primarily assessed patients

During the study period, 88 patients were contacted and screened for inclusion (see Fig. 1). Fifty-seven of the eligible patients (65%) could be included. Thirty (34%) declined to participate. Mainly this was due to a lack of interest in or fear concerning VR. After randomization into the sequence A/B (*n* = 28 ; SOC first, VR second) or B/A (*n* = 29 ; VR first, SOC second) 17/28 (61%) and 21/29 (72%) could be analyzed. 19/57 (33%) were not included in the final analysis as drop-outs or lost-to-follow-up. Reasons for refusal to participate in the study were documented (health reasons, no interest in glasses and/or study, Fear of glasses in intervention context, no problem with anxiety/pain, difficult conditions due to language barriers, finding an appointment for informed consent and observation). Uncertainty about the use of VR during a puncture, lack of interest in the study, or health reasons (worsening of health) were primary concerns here.

### Demographics

**Table 1:** Demographic and disease characteristics of the finally analyzed participants (*n* = 38)

	*n* (%)
Average age (median age) in years	11.5 (12.0)
Sex • Male • Female	19 (50%) 19 (50%)
Disease • Leukemia • Central nervous tumor • Lymphoma • Bone tumor • Other tumor • Thalassemia • Other anaemia	10 (26%) 3 (8%) 3 (8%) 2 (5%) 5 (13%) 11 (29%) 4 (11%)
Catheter used for intervention • Port catheter • Peripheral intravenous catheter • Broviac catheter	21 (55%) 15 (40%) 2 (5%)
Analgesia: use of local anesthetic cream in total • Port puncture with local anesthetic cream • Peripheral vein puncture with local anesthetic cream	20/38 (53%) 16/21 (76%) 4/15 (27%)

Out of the 38 patients evaluated after completed crossover, 50% were male and 50% were female (see Table 1). The average patient age was 11.5 years with a range of 6–18 years. The most common underlying diseases were leukemia (26%) and thalassemia (29%). The catheters used for the intervention were predominantly port catheters (55%). Patches with local anesthetics were used by 53% of the patients before skin punctures, whereas most of them were undergoing a port puncture (see Table 1). Characteristics such as age, sex or disease were similar for patients who declined study participation (data not shown).

### Peri-interventional use of VR and adverse events

76% of the patients chose to wear the VR glasses for the entire duration of the procedure, including both preparation and the puncture itself. The remaining 24% opted to use the VR glasses only during the preparation phase, removing them once the staff had prepared everything for the puncture. The time spent wearing the VR glasses varied depending on the individual treatment procedures, with some patients wearing them for as little as 2 min and in very few cases of bloodletting for up to 60 min, averaging at around 11 min. Adverse events occurred only rarely during use of the VR goggles. There were no side effects in almost 90% of the cases. Around 5% of the patients decided to discontinue the VR goggles prematurely due to discomfort. In individual cases, slight dizziness and nausea were reported, which, however, could not be attributed directly to the use of the VR goggles.

### Previous experience and stress management

**Fig. 2:**
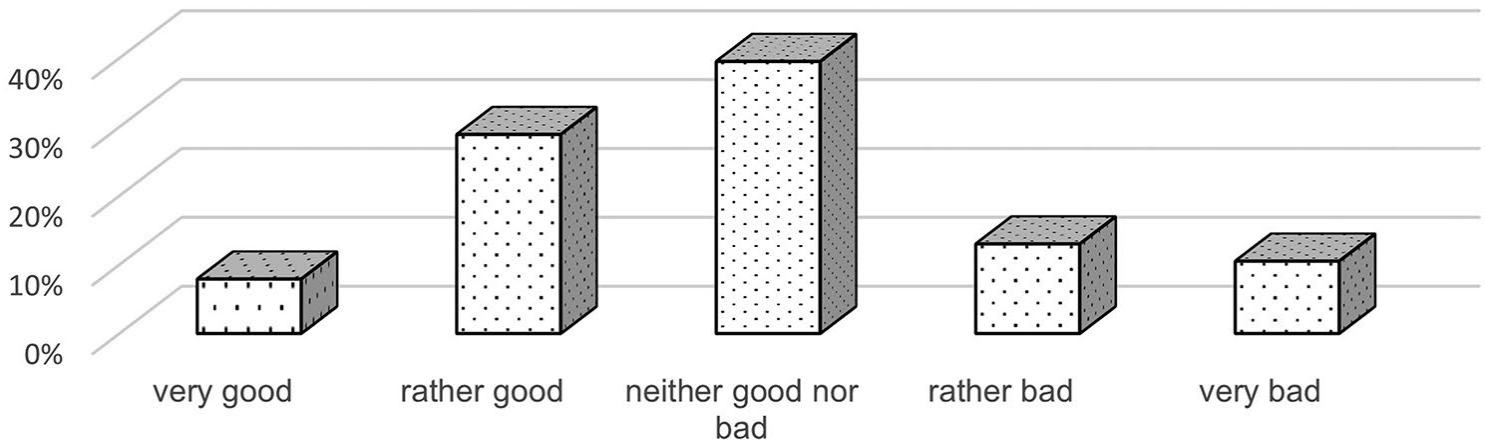
Prior experiences with the painful intervention measured with 5-point likert scale (*n* = 38)

Patients were asked about their previous experiences with the painful puncture and VR before taking part in the study. This was measured with the help of a 5-point likert scale. The experiences with the painful intervention were predominantly neither good nor bad. Figure 2 shows that the majority of study participants had similar attitudes towards painful punctures before taking part in the study. Most of the patients (61%) didn’t have any previous experience with VR. Previous distraction methods used were predominantly “none” (63%), the use of smartphone or tablet (18%) and distraction by parents (16%).

Off the detailed survey forms issued (self-report), 26 were returned. In terms of stress vulnerability, the majority of the SSKJ questionnaire showed average stress experience. Individual patients showed an above-average (very vulnerable) or below-average (very resilient) stress experience.

### Peri-interventional pain outcomes

**Fig. 3:**
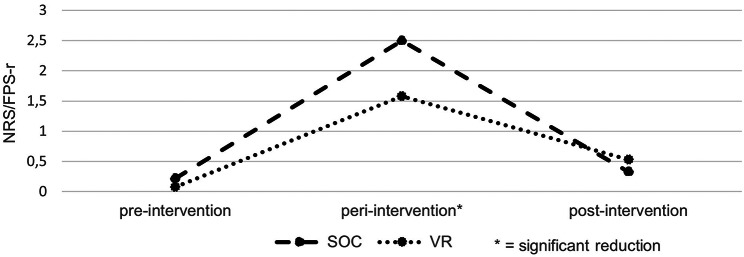
Pain outcomes measured with FPS-r/NRS (scale range: 0–10; *n* = 36)

Pain levels were documented before, during and after the potential painful intervention using FPS-r (6–9 years) and NRS (10–18 years). This was done for every painful puncture, two patients using a broviac catheter were not included here. While pain levels are very low before and after the intervention, there is a rise in pain perception peri-interventionally. Especially in group A (SOC) this rise is visible: the average pain changes from 0.22 to 2.5 to 0.33. In group B (VR) the pain is significantly lower peri-interventionally (*p* < 0.05): it changes from 0.08 to 1.58 to 0.53 (see Fig. 3). This shows patients seem to experience significantly lower levels of pain using the VR goggles during a painful puncture.

### Distress and anxiety outcomes

**Fig. 4:**
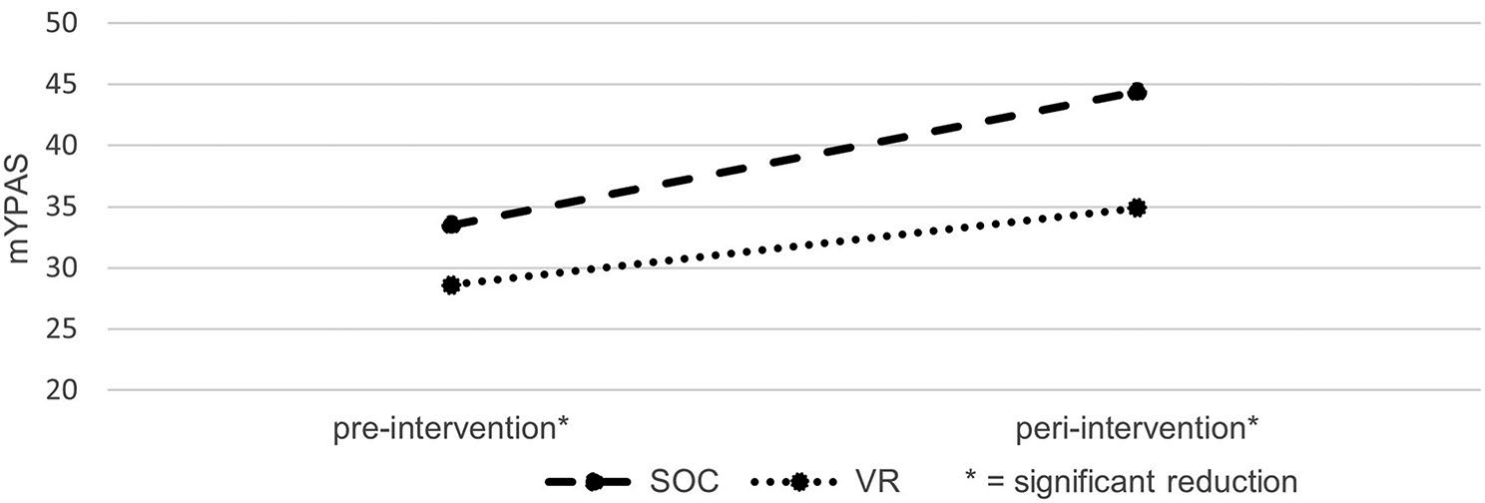
Anxiety outcomes measured with mYPAS-SF (scale range: 23–100, higher range stating higher levels of anxiety; *n* = 38)

Distress was documented using BAADS, whereas Anxiety was documented with help of mYPAS-SF. Both scales were used when the patient was entering the intervention room and just before/during the potentially painful intervention. For distress the sum of BAADS showed a significant reduction while using the VR-goggles in comparison to SOC (average of 2.6 in the VR-group vs. 3.1 for SOC; scale range 0–10; *p* < 0.01). Anxiety was measured separately for the two points in time, showing significantly lower levels (average of 28.6 in the VR-group vs. 33.5 for SOC; scale range 23–100) even before the actual usage of the goggles (*p* < 0.001). Peri-interventionally this reduction (average of 34.9 in the VR-group vs. 44.4 for SOC) is even more apparent (*p* < 0.001; see Fig. 4). This shows patients seem to experience significantly lower levels of anxiety while using VR goggles during a painful puncture.

### Subjective observations of patients and parents

**Fig. 5:**
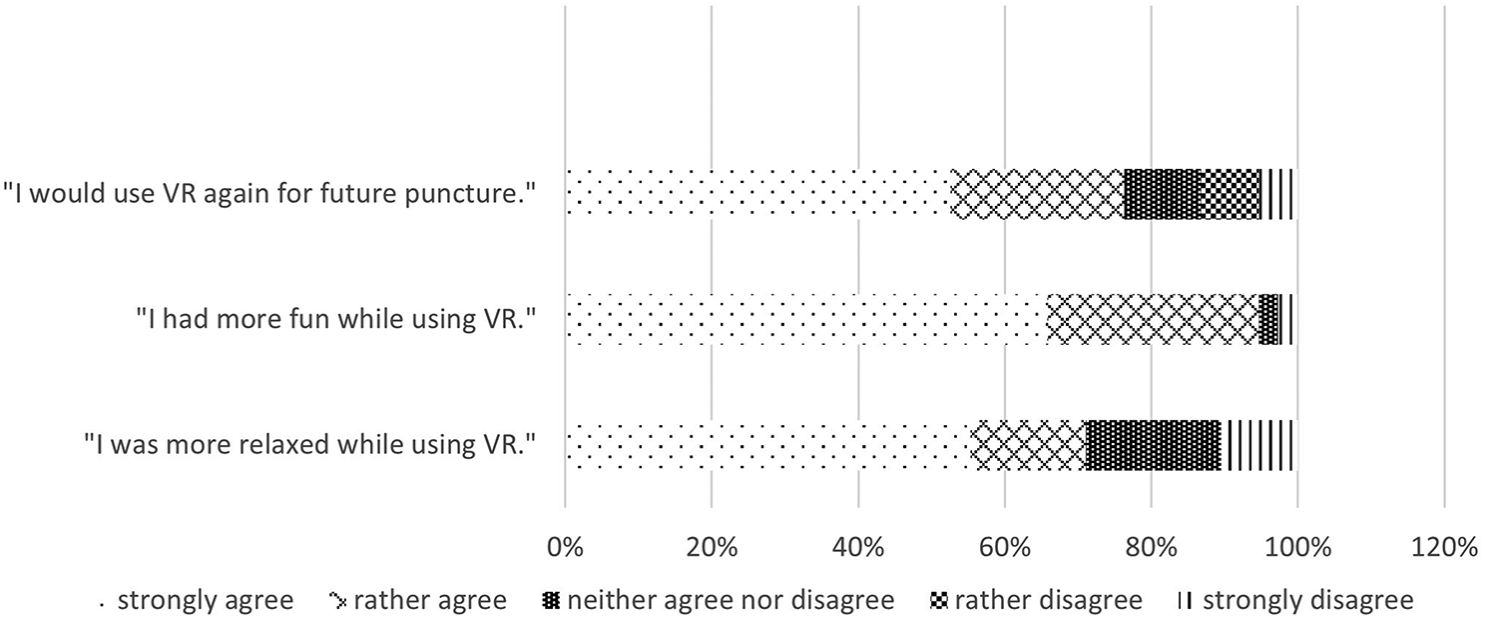
Observations on VR-usage from the patients perspective measured with 5-point likert scale (*n* = 38)

Relaxation and fun were measured using a 5-point likert scale. Most patients stated they felt more relaxed with VR accompanying the painful intervention. More than 90% claimed they had a fun experience using the VR-goggles. Nevertheless a few patients also stated they wouldn’t use VR again, because they didn’t feel more relaxed than usually (see Fig. 5).The parents perspective shows,, equivalent to the patients point of view, that there is a high agreement with relaxation through and future usage of VR. This was also measured using a 5-point likert scale.

### Hints for the implementation of VR

For the successful implementation of VR as a distraction method in an outpatient clinic setting, six main categories were identified. These include finance, responsibility, expertise, time, teamwork and use of VR. As a result of an interprofessional focus group discussion at the end of the study period, the following 10 tips were formulated by consensus of the group.1. Financial means for the acquisition and maintenance of the VR-equipment must be ensured. 2. Applicability should be considered in the choice of equipment (e.g. comfortable to wear for children, disinfection option). 3. When implementing VR in the outpatient clinic, nurses and physicians must be involved together. 4. The storage location and access to the VR goggles have to be coordinated within the team. 5. Responsibilities must be in few people. 6. Medical students and volunteers are suitable as additional staff for explaining VR to patients. 7. VR provider must be trained to avoid overstraining in stressful situations and technical problems. 8. Sufficient time must be planned for explaining the goggles when they are used for the first time. 9. Parents should be involved in the demonstration and explanation (role model function). 10. Evaluations of the application should take place regularly in order to quickly identify everyday problems.

## Discussion

### Peri-interventional use of VR as an approach to reduce pain and anxiety

The present study investigated the peri-interventional impact of VR as a distraction tool on the parameters of pain, anxiety, and distress in a pediatric oncology outpatient clinic. The effect of distraction during potentially painful examinations in general and the influence of VR in particular are well studied [15, 16]. While the quality of previous studies on pediatrics is heterogeneous, there is a lack of studies on its use in pediatric oncology outpatient clinics with corresponding investigation of its applicability in this specific setting [6]. The present study shows that the peri-interventional use of VR can decrease pain perception and pain-related anxiety. This could also be shown by Gerçeker et al. [17] in pediatric oncology patients for port needle-related anxiety and pain. A limitation is the high rate of refusals to participate in the study and the number of drop-outs or lost-to-follow-ups. Comparable challenges were also reported by Gold et al. [4]. Reasons could be skepticism about the glasses, short time intervals in the outpatient clinic, or insufficient information. Fear of adverse side effects or concern about loss of control could also contribute. This contrasts with the large proportion of subjectively positive experiences. Only 5% discontinued the glasses prematurely, whereas 95% expressed fun uing VR peri-interventionally. In the future, patients should be identified in advance who might particularly benefit from VR use or in whom an effect might produce undesirable effects. Questionnaires or screening instruments have not yet been developed for this purpose. The studies on distress and anxiety showed a significant reduction by VR. The clinical relevance is expressed in an increasing cooperativeness of the patients, which is recorded by the scores. This allows an easier procedure not only for patients but also for their parents and the staff involved. In this setting, the individual’s previous experiences and needs with regard to distraction methods, anxiety and stress should be given greater consideration in the patient’s medical history in order to make more room for the patient’s needs.

### Requirements in the implementation of VR in an outpatient clinic

A novel feature is the description of specific implementation instructions by an interprofessional focus group. Tennant et al. also focused on health care professionals. This research method can also be used to uncover local resistance and address information needs in the implementation process. Tennant et al. also focused on VR and its applicability [18]. There is disagreement as to which professional group should best bring VR to children. This is consistent with our findings that new personnel and time resources need to be made available. The additional time required of existing staff or the need to employ additional, well-trained staff proved challenging. A “low-threshold” introduction of VR glasses as a distraction method does not appear to make sense due to content and technical challenges. Therefore personnel and time resources must be made available to ensure effective support in terms of individually taking care of patients, their families and also the technical requirements.

### Limitations and future research

One weakness of the study is the number of drop-outs and refusals to participate in the study. Reasons for this included uncertainty about the use of VR for punctures or a lack of interest in participating in the study. Among the observations that were not completed, the main reasons were patients’ health and difficulties in scheduling. This seems to be a realistic reflection of complex clinical situations. A higher number of researchers would have been advantageous here in terms of inclusion, but challenging for the standardization of observations. Gold et al. also mention comparable numbers of participants and refusals, which supports these speculations [4].

A limitation, in addition to the presented rate of fully completed cross-over observations, is the influence of the investigators due to the required observations for scores and scales. Objective measurements of the examined parameters are difficult in the clinical routine and in the outpatient clinic. Extensive training of the investigators should reduce a corresponding bias. The limited response of detailed questionnaires to describe stress management in the collective reduces the validity. A reduced number of survey items could increase the response rate but limits the breadth of information generated. In addition, it would be conceivable to examine patients without any previous experience. Patients with no previous experience of the procedure were not included in the study group, meaning that the effect on completely naïve patients cannot be assessed with certainty. Previous experience was recorded using a Likert scale from the important patient perspective (see Fig. 2), but without correlating it with the exact number of previous punctures. Future investigations of receptivity to VR as a distraction method should target personal factors and capture experiences as a baseline for choosing the best support.

Observation time points peri-interventionally were limited to entering the room, the moment immediately before the intervention, and a few minutes after the intervention. Video documentation with subsequent close-meshed analysis could be used - but in a very resource-intensive way - for a more detailed description. Bias due to retrospective recording of patient, parent, and staff experiences are conceivable.

Studies suggest the potential to reduce medications. Nevertheless, anti-anxiety and analgesic medications should not be withheld from any patient immediately for bone marrow puncture. The primary purpose of VR goggles is to increase the quality of treatment. Due to the limited number of cases, the subgroup analyses could not provide any meaningful, clinically relevant results regarding differences in the effect of VR in subgroups. All options for reducing pain, anxiety and stress should be considered for the individual patient.

Although technological capabilities expand the range of diversionary methods, the primary focus remains on trained, attentive, and flexible staff who must anticipate the best way to provide peri-interventional support as conditions change and patients vary individually.

Future studies need to shed light on the influence of VR as a complementary aspect of successful distraction methods peri-interventionally and provide impetus to identify particularly susceptible patients. Nevertheless, even the consideration of VR as a method of distraction for pain and stress reduction can lead to a meaningful discussion of the topic in terms of improving patient care.

## Data Availability

The datasets used and analysed during the current study are available from the corresponding author on request.
